# Marker chromosomes in 15 individuals: cytogenetic delineation and molecular findings from SNP array analysis in a subset of cases

**DOI:** 10.1186/s13039-026-00777-0

**Published:** 2026-08-11

**Authors:** Minjun Huang, Jiexiang Huang, Qingxiang Chen, Lifang Wang, Kun Lin

**Affiliations:** 1 Prenatal Diagnosis Center, Affiliated Hospital of Putian University, Putian University, Putian, 351100 Fujian Province China; 2 Newborn Screening Center, Putian Maternity and Child Health Care Hospital, Putian, 351100 Fujian Province China

**Keywords:** Marker chromosome (MCs), Karyotype analysis, SNP array, Prenatal diagnosis, Genetic counseling

## Abstract

**Objective:**

Marker chromosomes (MCs) are structurally abnormal chromosomes that can be detected by standard cytogenetic banding techniques but their precise structure and origin cannot be fully determined by these methods alone. These abnormal chromosomes can be present as supernumerary chromosomes in an otherwise normal karyotype or replace a normal chromosome in a structurally abnormal karyotype. MCs can originate from any of the 24 chromosomes, which makes genotype-phenotype correlation a challenge in clinical diagnosis and genetic counseling for MCs carriers. In this study, we combined cytogenetic and molecular diagnostic techniques to determine the origin of MCs. By correlating the associated genomic variations with clinical phenotypes, we aimed to provide preliminary evidence-based medical guidance for individuals carrying MCs.

**Methods:**

We retrospectively reviewed 15 cases of marker chromosomes (MCs) identified by conventional cytogenetic testing among 9,733 individuals at our center. A subset of these cases was further analyzed by single nucleotide polymorphism (SNP) array to determine the precise origin, size, and genomic content of the MCs.

**Results:**

In this cohort, 15 carriers of marker chromosomes (MCs) were identified, including 13 with mosaic and 2 with non-mosaic karyotypes. Seven of these patients underwent molecular analysis. Of these, SNP array revealed that five carried MCs derived from the X/Y chromosomes. In one case, a duplicated fragment at 11p11.12q12.2 was identified, whereas in another, no copy number variations (CNVs) were detected by SNP array. The clinical phenotypes of the eight postnatal MC carriers were diverse, ranging from asymptomatic to severe growth and developmental delay. Observed manifestations included short stature, primary amenorrhea, and infertility.

**Conclusion:**

Marker chromosomes (MCs) exhibit substantial genetic and clinical heterogeneity. The combined use of karyotyping and SNP array analysis effectively determined their chromosomal origin and facilitated the assessment of their pathogenic potential. Clinical outcomes were found to be strongly associated with the structure, chromosomal origin, and mosaic ratio of the MCs. This integrated diagnostic approach enables more accurate genotype-phenotype correlation and facilitates informed genetic counseling for affected individuals.

## Introduction

Marker chromosomes (MCs) are structurally abnormal chromosomes that cannot be unambiguously identified or characterized by conventional banding cytogenetics [1]. MCs often contain a centromere, rendering them cytogenetically visible through standard karyotyping. Despite their detectability, the genomic content and clinical relevance of MCs frequently remain unclear [2]. MCs can originate from any of the 24 human chromosomes (22 autosomes and two sex chromosomes), and due to their heterogeneous genetic content, their phenotypic effects range from benign to pathogenic. This variability poses a significant challenge in genetic counseling, prenatal diagnosis, and clinical management—particularly when the chromosomal origin and gene content of the marker are not well-defined [3, 4].

The clinical significance of MCs depends on several critical factors: their chromosomal origin, the presence of dosage-sensitive genes within the duplicated region, the degree of mosaicism (i.e., the proportion of cells carrying the MCs), and whether the individual is an asymptomatic carrier or presents with clinical symptoms [5, 6]. For example, MCs derived from chromosome 15 are among the most frequently reported and are associated with well-characterized syndromes such as Prader-Willi and Angelman. Conversely, some MCs are inherited from phenotypically normal parents and may not cause any observable clinical manifestations. This complex and sometimes unpredictable relationship between genotype and phenotype necessitates cautious interpretation. Such interpretation is often supported by advanced molecular diagnostic methods [7, 8].

Historically, the characterization of MCs was constrained by the limited resolution of conventional cytogenetics. However, the development of molecular cytogenetic techniques, including fluorescence in situ hybridization (FISH), array comparative genomic hybridization (aCGH), and more recently, single nucleotide polymorphism (SNP) arrays, has substantially improved our ability to determine the chromosomal origin, size, and genomic content of MCs. Among these, SNP arrays offer high-resolution data on copy number variations (CNVs) and uniparental disomy, both of which are critical for evaluating the clinical implications of MCs [9]. Despite these technological advances, large-scale, systematic studies investigating correlations among chromosomal origin, mosaicism, and clinical phenotype are still lacking. Much of the existing literature comprises case reports or small case series, limiting generalizability and hindering the establishment of robust genotype–phenotype relationships [10]. Additionally, the potential impact of low-level mosaicism is frequently underestimated in clinical settings, even though it may significantly influence disease severity and phenotypic variability [11–13].

By examining the relationship among MCs origin, mosaic status, and clinical phenotype, this study aims to provide deeper insights into the interpretation of MCs in both prenatal and postnatal settings. Our results may inform more precise genetic counseling, guide early clinical interventions, and contribute to the development of individualized care strategies based on molecular features. Ultimately, this work underscores the critical role of comprehensive, high-resolution genomic testing in the diagnostic evaluation of individuals with suspected chromosomal abnormalities, particularly when MCs are detected.

## Materials and methods

### Sample collection

This was a retrospective study included 9,733 cytogenetic samples referred to Prenatal Diagnosis Center of the Affiliated Hospital of Putian University between January 2020 and December 2023. Cytogenetic testing was performed for individuals with clinical or prenatal indications such as abnormal screening results, ultrasound findings, infertility, recurrent miscarriage, or developmental delay. Among these, 15 cases carrying marker chromosomes (MCs) were identified by conventional G-banding analysis, comprising 8 prenatal samples (amniotic fluid cells) and 7 postnatal cases (peripheral blood samples). All patients were counseled regarding further parental origin testing and SNP array. SNP array was ultimately performed in 7 cases, and parental origin testing was available in 8 cases. Informed consent was obtained from all participants or their legal guardians.

### Cytogenetic analysis

All samples underwent conventional G-banding karyotype analysis following standard procedures for peripheral blood or amniotic fluid culture and chromosome preparation. Chromosome preparations were treated with trypsin and stained with Giemsa (G-banding). Metaphase spreads were then scanned using a Leica GLS120 automated scanning system, and images were captured and analyzed with Cytovision software. A minimum of 20 metaphase spreads was analyzed per case. In cases with high suspicion of mosaicism, or where evidence suggested low-level mosaicism, the number of cells analyzed was increased in accordance with established guidelines [14]. Karyotypes were described according to the International System for Human Cytogenomic Nomenclature (ISCN 2024).

### Detection of Y-chromosome microdeletions

Genomic DNA was extracted from peripheral blood lymphocytes (fresh or frozen, collected in 2 mL EDTA tubes) using a commercial kit (Da’an, Guangzhou) according to the manufacturer’s instructions. Y-chromosome AZF microdeletions were analyzed with a fluorescent probe-based PCR detection kit (Tellgen, Shanghai) targeting six sequence-tagged sites (STS) located in the AZFa, AZFb, and AZFc regions. Two internal control genes were included: *SRY* (male-specific) and *ZFX/ZFY* (encoding zinc finger proteins). The STS markers covered AZFa (sY84, sY86), AZFb (sY127, sY134), and AZFc (sY254, sY255).

### Molecular analysis (SNP array)

Seven out of the 15 cases underwent further analysis using SNP array to determine the chromosomal origin, fragment size, and pathogenicity of the MCs. Genomic DNA was extracted using the Magnetic Universal Genomic DNA kit (TIANGEN Biotech, Beijing) for amniotic fluid and MagaBio plus General Genomic DNA Purification Kit II (BORI Biotech, Hangzhou) for peripheral blood, and analysis was performed on the Affymetrix CytoScan 750 K Array platform. Data were processed using Chromosome Analysis Suite (ChAS4.5) software (Thermo Fisher Scientific Inc.), and genomic positions were annotated based on the human genome reference build GRCh37. The reporting thresholds for CNVs were set at 50 kb with marker count ≥ 25 for losses, 100 kb with marker count ≥ 25 for gains, and 5 Mb with marker count ≥ 50 for absence of heterozygosity (AOH).

Regarding mosaic detection, the platform can reliably detect mosaic copy number changes at levels above approximately 30%, while mosaicism in the 10–30% range may be detectabe in samples with good-quality data.

### CNVs classification and pathogenicity assessment

The clinical significance of candidate CNVs was interpreted and classified using multiple databases, including DECIPHER, ClinVar, OMIM, and ClinGen, in accordance with previously reported guidelines [15, 16]. All identified CNVs were categorized into five classes (pathogenic, likely pathogenic, VOUS, likely benign, and benign) based on the American College of Medical Genetics and Genomics (ACMG) standards. Subsequently, the structural characteristics of these CNVs were correlated with clinical phenotypes to assess potential genotype-phenotype relationships.

### Pregnancy outcomes and clinical follow-up

For prenatal cases, pregnancy outcomes were collected from clinical records and follow-up, including termination of pregnancy or postnatal health status. For postnatal individuals, clinical phenotypes were assessed by physicians through medical history, physical examination, and ancillary investigations.

## Results

### Analysis of the incidence and cytogenetic morphology of marker chromosomes across different samples

A total of 15 cases of MCs were detected among 9,733 samples in this study. Of these, 7 cases were detected in peripheral blood lymphocytes, with an incidence of 0.99‰ (7/7,088), and 8 cases were found in amniotic fluid samples from prenatal diagnoses, with an incidence of 3.02‰ (8/2,645) (Table 1). Among them, 13 cases of MCs were present in mosaic form, while only 2 were non-mosaic. Parental origin analysis of MC was performed in 8 cases; 2 were inherited from the mother, while 6 were *de novo*. SNP array was conducted in 7 cases: one carrying an MC showed no detectable CNVs, while CNVs were identified in the remaining 6 cases. Among these, 1 involved an autosome and 5 involved sex chromosomes, including 2 cases with Turner syndrome and 3 cases with Y chromosome segmental deletions. In addition, one case of Y chromosome microdeletion (case 6) was confirmed by PCR analysis.

Accurately determining the chromosomal origin of these MCs based on their cytomorphological features is difficult, as most are no larger than a chromosome 20 in the same metaphase spread. Case 7 was a notable exception. Despite the relatively large size of its MC, the banding pattern remained indiscernible. Consequently, without supporting molecular data, it was also classified as a marker chromosome. In peripheral blood samples, the MCs in cases 1, 2, and 4 were all deeply stained heterochromatin. In case 5, the MC contained a satellite body at the terminal region, suggesting a possible origin from an acrocentric chromosome. In cases 3, 6, and 7, the MCs exhibited alternating light and dark bands; however, due to their small size, their exact origin could not be visually determined. In amniotic fluid samples, MCs in cases 8 and 9 were morphologically more consistent with heterochromatin, while the remaining cases 10 to 14 all displayed alternating light and dark bands, and case 15 was morphologically consistent with euchromatin. However, due to their small size and the absence of concomitant molecular testing, the specific chromosomal origin of these fragments could not be accurately determined. Case 13 was the only ring-shaped marker chromosome identified in this study.

**Table 1:** Summary of MCs in this study

Category	Subcategory	Case number	Peripheral blood	Amniotic fluid		
Total samples		9733	7088	2645		
Cases carried with MCs		15	7	8		
Incidence by sample type			0.99‰	3.02‰		
Karyotype	Mosaic	13	6	7		
	Non-mosaic	2	1	1		
Origin of MCs	Maternal	2				
	*de novo*	6				
	Not tested	7				
SNP array results	Normal	1				
	Abnormal	6	Sex chromosome	5	Turner syndrome	2
					Y chromosome deletion	3
			Autosome	1		
Y chromosome gene microdeletions (PCR-based)		1 (Case No.6)				

Distribution of 15 cases identified from 9,733 cytogenetic samples, stratified by sample type (peripheral blood, amniotic fluid), karyotypes (mosaic, non-mosaic), parental origin (maternal, *de novo*, not tested), and SNP array results (sex chromosome abnormalities, e.g., Turner syndrome, Y-chromosome deletions; or autosomal abnormalities). Case 6 underwent PCR validation for Y-chromosome microdeletions. Data are presented as number of cases. Abbreviations: MC, marker chromosome; SNP, single nucleotide polymorphism

### Karyotype results and clinical features of seven patients with MCs detected in peripheral blood

Cases 1 to 7 represent postnatal subjects, and their clinical manifestations are summarized in Table 2. Among the postnatal population carrying MCs, most occurred in mosaic form. In cases 1, 2, and 4, the MCs were present in mosaic form as 47,XX,+mar (or 47,XY,+mar) with normal cellular background. Cytogenetic analysis showed the marker chromosomes were composed predominantly of intensely stained heterochromatin. Case 1 exhibited a normal phenotype and has delivered one child via cesarean section; case 2 shows normal growth and development to date; case 4 presented solely with short stature, in the absence of intellectual disability or psychobehavioral abnormalities. Specifically, cases 3, 6, and 7 displayed Turner syndrome–type MCs karyotypes, i.e., 45,X/46,X,+mar. Case 6 is a 48-year-old male who underwent surgery for hypospadias over 30 years ago and was hospitalized for recurrent hematuria. His karyotype was 45,X[93]/46,X,+mar[7]. Further PCR-based analysis revealed absence of the AZFb region on the Y chromosome. Scrotal ultrasonography showed no identifiable testicular tissue in either hemiscrotum; however, pelvic imaging revealed a mass suggestive of a seminal vesicle tumor. Two remaining pediatric cases (Case 3 and 7) exhibited clinical features of short stature and developmental delay. Case 5 was confirmed by parental testing to be maternally inherited. Among the seven postnatal cases, only case 7 underwent SNP array.

**Fig. 1:**
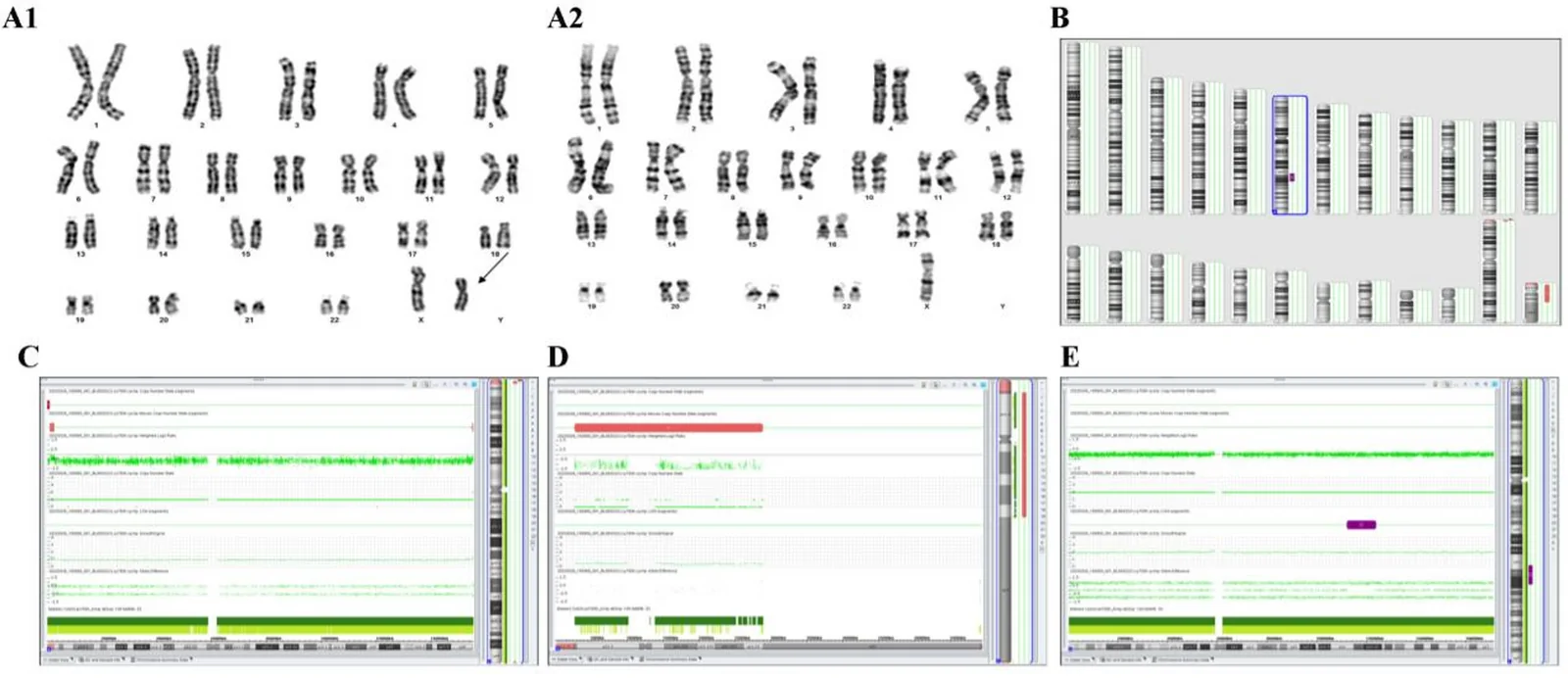
Cytogenetic and SNP array results of marker chromosome in case 7. (**A1, A2**) G-banding of case 7 revealed the karyotype 45,X[16]/46,X,+mar[26]; (**B**) Whole chromosome ideogram of the patient, where red represents deletions in X and Y chromosome (Loss) and purple represents AOH in chromosome 6; (C, D) The MC of case 7 was a partial mosaic of deletions in chromosomes X and Y, with a ratio of 0.68. (**C**) shows a detailed view of the deletion in chromosome X, while (**D**) shows the deletion in chromosome Y (**E**) Detailed view of the AOH region on chromosome 6 in case7, arr[GRCh37] 6q21q22.31(111,919,462 − 124,089,069)x2 hmz

Case 7 is a 15-year-old phenotypic female with short stature, growth retardation, borderline intellectual impairment, and primary amenorrhea. Abdominal ultrasound failed to identify a normal uterine configuration, revealing only a strip of hypoechoic area was observed posterior to the bladder. Her karyotype was 45,X[16]/46,X,+mar[26]. Based on her clinical phenotype, we hypothesized that the MC originated from the sex chromosomes, a finding that was subsequently confirmed by molecular testing. Unexpectedly, the SNP array results indicate a male, and the results of SNP array were summarized in Table 3. These results indicate the marker chromosome is a pseudo-isodicentric Y chromosome [psu idic(Y)(p11.32)], and the correct karyotype is 45,X[16]/46,X, psu idic(Y)(p11.32)[26]. The formation of this pseudo-isodicentric Y chromosome likely resulted from a U-type sister chromatid exchange during meiosis or early post-zygotic mitosis. This event led to the duplication of Yp and the proximal Yq region, accompanied by the deletion of the distal Yq segment. Additionally, SNP array revealed a 12.1 Mb region of absence of heterozygosity (AOH) at 6q21–q22.31, without copy number change (Fig. 1E), encompassing nine autosomal recessive disease associated genes such as *CCN6*,* TSPYL1*, and *DSE*. The clinical relevance of this absence of heterozygosity remains unclear. Unfortunately, parental analysis was not possible: the father is deceased, and the mother declined further familial testing, so it is unknown whether the AOH of 6q is uniparental disomy or inherited.

### Clinical information and molecular diagnostic results of 8 fetuses carrying MCs in amniotic fluid samples

Cases 8 to 15 are prenatal fetal amniotic fluid samples. The indications for prenatal diagnosis of the pregnant women and fetuses, chromosomal karyotypes, and SNP array results are shown in Table 4.

**Fig. 2:**
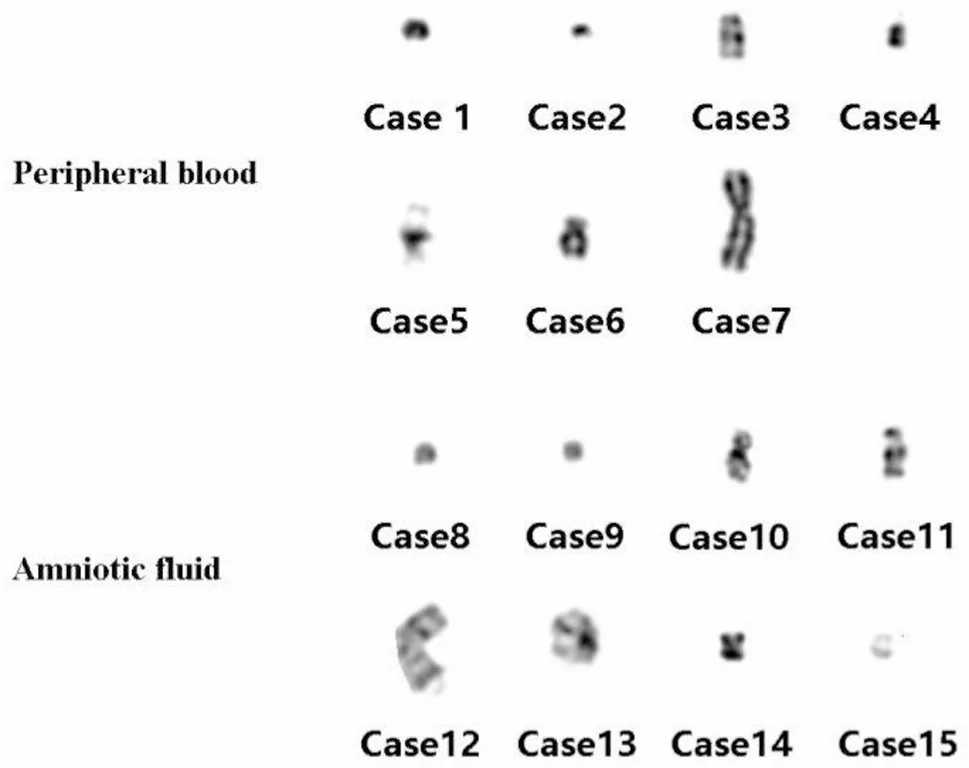
Cytogenetic morphological characteristics of marker chromosomes in 15 cases

Notably, in case 10, chromosomal karyotype analysis revealed that more than 95% of the amniotic fluid cells carried MCs, with morphology shown in Fig. 2 (Case 10). Parental verification confirmed that this MCs was *de novo*. However, SNP array did not detect any CNVs. A live female infant was delivered, and she is now 2 years old with normal growth parameters. Continued follow-up is in progress.

Case 15 was also a *de novo* MC. SNP array identified a ~ 10.0 Mb contiguous gene duplication at 11p11.12q12.2, which was classified as a likely pathogenic (LP) variant. This genomic imbalance, resulting in the dosage increase of multiple genes including *CTNND1* and *FAM111B*, is the postulated pathogenic mechanism. Cases have been reported of smaller duplications of this fragment being associated with clinical phenotypes including downslanting palpebral fissures, autism, and deep-set eyes (DECIPHER Patient: 265194, Uncertain), as well as obesity, specific learning disabilities, and macrocephaly (DECIPHER Patient: 379332, Uncertain). In this prenatal case, aside from bilateral hydronephrosis detected by ultrasound, no other sonographic anomalies were observed. After thorough counseling regarding the potential risks associated with this likely pathogenic finding, the family opted for pregnancy termination.

Combining the SNP array results, it was found that most MCs detected in prenatal fetal amniotic fluid originated from structural abnormalities of sex chromosomes (X/Y), accounting for 83% (5/6) in this study. These included psu idic(Y)(Case 11), del(X)(Case 12), r(X)(Case 13), and del(Y)(Case 14). Large deletions in these chromosomes obscured their characteristic banding patterns, and SNP array helped confirm the origins of these marker chromosomes, providing more accurate guidance for clinical prenatal genetic counseling.

In case 13, conventional cytogenetic G-banding of fetal amniotic fluid cells showed a karyotype of 45,X[47]/46,X,+r(mar)[13] dn, representing a *de novo* ring MCs and mosaicism with monosomy X(45,X). SNP array results revealed mosaic findings in the fetus. The predominant cell line was 45,X, accounting for approximately 70% of cells. A second cell line, present in approximately 30% of fetal cells, showed heterozygous deletions of 27.6 Mb at Xq25q28 and 54.1 Mb at Xp22.33p11.22. The Xq25q28 deletion encompasses 192 OMIM genes, including *MTCP1*, *BRCC3*, and *MECP2*. Pathogenic variants in *MTCP1* and *BRCC3* are associated with X-linked recessive Moyamoya disease 4, while loss of *MECP2* causes X-linked dominant Rett syndrome. Associated clinical phenotypes include language delay, autism, and intellectual disability. The Xp22.33p11.22 deletion contains 288 OMIM genes, notably *SHOX* and *STS*. *SHOX* is critical for skeletal development and is clearly haploinsufficiency, while deletion of *STS* causes X-linked recessive ichthyosis. These variants were classified as pathogenic. Based on the SNP array findings, the marker chromosome was characterized as r(X)(p11.22q25).

**Table 2:** Karyotype Analysis of Peripheral Blood in 7 Cases

Cases No.	Sex/Age	Karyotype	Clinical symptoms	MC morphology	de novo/ inherited
1	female/29y	47,XX,+mar[36]/46,XX[64]	History of one missed abortion	Figure2. case1	—
2	male/2d	47,XY,+mar[81]/46,XY[24]	Premature infant, DS cut-off risk, NIPT failed	Figure2. case2	—
3	male/6y	45,X[10]/46,X,+mar[90]	Short stature, developmental delay H:107 cm, W:16.6 kg	Figure2. case3	—
4	female/5y	47,XX,+mar[33]/46,XX[47]	Short stature, H:108 cm, W:20 kg	Figure2. case4	—
5	female/26y	47,XX,+mar mat	Insulin resistance, infertility	Figure2. case5	maternal
6	male/48y	45,X[93]/46,X,+mar[7]	Hematuria for 3 months, Pelvic gonadal tumor. He had surgery 30 years ago for congenital hypospadias	del(Y)	—
7	female/15y	45,X[16]/46,X,+mar[26]	Short stature, H:136.4 cm(-3SD), intellectual disability. Ultrasound revealed no uterus or ovaries; however, a hypoechoic band was noted posterior to the bladder.	psu idic(Y)	—

Cytogenetic and clinical characteristics of seven peripheral blood cases with marker chromosomes (MCs). Data include gender, age, karyotypes (mosaic/non-mosaic proportions), clinical manifestations, MC morphology (cytogenetic/molecular), and parental origin. Symptoms are per medical records; mosaicism is percent analyzed metaphases

MC, marker chromosome; mat, maternal origin; idic, isodicentric chromosome; del, deletion; DS, Down syndrome; NIPT, non-invasive prenatal testing; H, height; W, weight

**Table 3:** Summary of SNP array results in case 7

SNP array	Size	PC	Description
Xp22.33 or Yp11.32(168,552-1,040,206 or 118,552–990,206)×1	871.6 kb	P	A 871.6 kb deletion in the pseudo-autosomal region of the short arm, encompassing five OMIM genes including *SHOX/SHOXY*, important for skeletal development and known to exhibit haploinsufficiency associated with short stature
Xp22.33 or Yp11.32p11.31(1,101,626-2,693,467 or 1,051,626-2,643,467)×1–2	1.5 Mb	VOUS	In approximately 68% of cells, a 1.5 Mb deletion was detected in the pseudo-autosomal region of the short arm
Xq28 or Yq12 (154,941,869 − 155,233,098 or 59,044,875 − 59,336,104)×1–2	291.2 kb	VOUS	A 291.2 kb deletion in the pseudo-autosomal region of the long arm was observed in about 68% of cells
Yp11.31q11.23(2,650,425 − 28,799,654)×0–1	26.1 Mb	P	A 26.1 Mb deletion spanning Yp11.31–Yq11.23 was present in approximately 68% of cells
6q21q22.31(111,919,462 − 124,089,069)×2 hmz	12.1 Mb	VOUS	A 12.1 Mb region of absence of heterozygosity (AOH) at 6q21–q22.31, without copy number change, encompassing nine autosomal recessive disease–associated genes such as *CCN6*,* TSPYL1*, and *DSE*. The clinical relevance of this loss of heterozygosity remains unclear.

**Table 4:** SNP Array Results and Clinical Findings in Amniotic Fluid from 8 Fetuses Carrying MCs

Cases No.	Maternal age	Clinical symptoms	Karyotype of amniotic fluid cells	SNP array	Size	PC	MCs morphology	outcome
8	22y	High risk of DS (T21,1/184)	47,XX,+mar[57]/46,XX[47] mat	—	—	—	—	Caesarean section
9	29y	High risk of DS (T21,1/97)	47,XY,+mar[11]/46,XY[92]	—	—	—	—	Caesarean section
10	38y	History of two spontaneous abortions; advanced maternal age	47,XX,+mar[95]/46,XX[5] dn	(1–22)×2, (XN)×1		Normal		Natural childbirth
11	27y	NIPT: high risk of sex chromosome aneuploidy	45,X[58]/46,X,+mar[42] dn	Yp11.31q11.222(2,650,424 − 20,810,603)×0–1	18.1 Mb	P	psu idic(Y)	Terminated
				Yq11.222q11.23(20,810,604 − 28,799,654)×0	7.9 Mb	P		
12	31y	NIPT: abnormal X and 7 chromosomes	46,X,+mar dn	Xq21.33q28(94,357,778 − 155,233,098)×1	60.8 Mb	P	del(X)	Intrauterine fetal death
13	25y	NIPT: high risk of sex chromosome aneuploidy	45,X[47]/46,X,+r(mar)[13] dn	Xq25q28(127,620,308 − 155,233,098)×1	27.6 Mb	P	ring(X)	Terminated
				Xp11.22q25(54,410,925 − 127,592,679)×1–2	73.1 Mb	P		
				Xp22.33p11.22(168,552 − 54,272,026)×1	54.1 Mb	P		
14	30y	Ultrasound showed that right-sided aortic arch with mirror-image branching and a left-sided ductus arteriosus.	45,X[48]/46,X,+mar[52] dn	Yq11.22q12(19,297,292 − 28,819,361)×0	9.52 Mb	P	del(Y)	Terminated
				Yp11.32q11.22((10,001–19,297,292)×0–1	19.29 Mb	P		
15	28y	Ultrasound showed that fetal bilateral hydronephrosis	47,XY,+mar[95]/46,XY[7] dn	11p11.12q12.2(50,477,716 − 60,508,898)×3	10 Mb	LP	dup(11)(p11.12q12.2)	Terminated

Cytogenetic and molecular findings of eight fetuses with marker chromosomes (MCs) from amniotic fluid. Data include maternal age, karyotypes (mosaic/non-mosaic proportions), SNP array results, CNV sizes, parental origin, clinical indications (e.g., NIPT-high risk, ultrasound findings), MC morphology, and pregnancy outcomes. CNVs are classified as normal, pathogenic (P), or likely pathogenic (LP). Abbreviations: MCs, marker chromosome; PC, pathogenicity classification; mat, maternal; dn, *de novo*; psu idic, pseudo-isodicentric; del, deletion; dup, duplication; r, ring; DS, Down syndrome; NIPT, non-invasive prenatal testing

## Discussion

Traditional cytogenetic G-banding can detect the presence of MCs but cannot determine their origin. Furthermore, the reported frequency of MCs varies considerably across different study populations [17, 18]. Moreover, phenotypic variability among MCs carriers is considerable, and the severity of clinical phenotypes is closely related to chromosome origin, gene dosage, size, degree of mosaicism, presence of uniparental disomy, and tissue distribution of MCs [19, 20]. In this study, we analyzed chromosomal karyotypes from 9,733 patients referred to a genetic center. The analysis included 7,088 peripheral blood samples and 2,645 prenatal amniotic fluid samples. We identified 15 MCs carriers, and among these, 7 cases underwent additional molecular testing using SNP array to determine the origin of MCs and assess genotype-phenotype correlations.

### Marker chromosomes in normal karyotypes

Among the 15 cases in this study, 8 cases had the karyotype 47,XX,+mar or 47,XY,+mar, including postnatal cases 1, 2, 4, and 5, and prenatal amniotic fluid cases 8, 9, 10, and 15. It can be seen that whether in postnatal individuals or prenatal amniotic fluid, the clinical manifestations of MCs present in the 47,XX,+mar or 47,XY,+mar karyotype are milder compared to MCs in Turner syndrome cases. Analysis of the morphology of these eight MCs showed that except for case 5, whose MCs is more likely derived from acrocentric chromosomes, the other 6 cases consisted of deeply stained heterochromatic small fragments. Although no further precise molecular genetic diagnosis was performed for case 5, the same MCs was also detected in the mother’s peripheral blood, and the clinical phenotype was normal. Studies have shown [21–23] that over 98% of inherited MCs carriers have normal clinical phenotypes; about 70% of *de novo* cases have no phenotype correlation with MCs. If MCs originate from chromosomes 13, 14, 21, or 22, the risk of abnormal phenotypes related to *de novo* MCs is approximately 7%, whereas if MCs originate from non-acrocentric chromosomes, the risk of causing abnormal phenotypes increases to 28%. In this study, cases 10 and 15 had MCs derived from non-acrocentric chromosomes. Case 10’s MCs was *de novo*, and further SNP array results showed no abnormalities. The fetus was delivered smoothly, and no abnormal clinical phenotype has been observed to date. Warburton D [24]. reported that the risk of abnormal fetal phenotype related to MCs in prenatal diagnosis is 13%, highlighting the importance of precise characterization of MCs for prenatal diagnosis and genetic counseling. However, information obtained through prenatal ultrasound imaging of the fetus is limited, especially for *de novo* MCs, making genotype-phenotype correlation determination extremely difficult prenatally. In case 15, the *de novo* MC was identified by SNP array as dup(11)(p11.12q12.2), with a duplicated fragment of about 10 Mb. According to genetic databases, this variant was classified as likely pathogenic. Consistent with comprehensive reviews on small supernumerary marker chromosomes derived from human chromosome 11, such MC(11) cases are exceedingly rare and exhibit significant phenotypic heterogeneity [25].

### Marker chromosomes in Turner syndrome cases

The incidence of Turner syndrome in second-trimester fetal amniotic fluid samples is 1.76 per 1,000, with the occurrence of MCs in Turner syndrome (MCs^T^) being 3.08% [26]. MCs^T^ carriers predominantly have the karyotype 45,X/46,X,+mar. MCs^T^ mainly present in three morphological forms: dicentric chromosomes (dic), ring chromosomes (r), and minute chromosomes containing centromeres (min). Almost all MCs^T^ have been confirmed to be derived from the X or Y chromosomes, with about 72.6% originating from the Y chromosome [27]. Most MCs^T^(Y) cases are isodicentric chromosomes, and MCs^T^(Y) patients have a significantly increased risk of gonadoblastoma, with gonadoblastoma and carcinoma in situ capable of progressing to non-invasive or invasive malignant germ cell tumors [28]. The remaining 27% of MCs^T^ are derived from the X chromosome, most of which are ring chromosomes [29]. A very small proportion (0.4%) originate from autosomes [30], potentially causing Turner syndrome phenotypes or gonadal dysgenesis.

In this study, case 6, a 48-year-old male, showed a pelvic mass on abdominal ultrasound. Y chromosome microdeletion PCR confirmed the absence of the AZFb gene region. Combining the Y microdeletion results, the patient’s karyotype was defined as 45,X[93]/46,X, del(Y)(q11.23)[7]. Although the 45,X cell line predominated (> 90%), the patient exhibited partial male secondary sexual characteristics, likely due to the presence of *SRY* and the fact that only part of the *AZFb* region was deleted. However, no significant testicular or epididymal echoes were detected in the scrota bilaterally. A uterus, right ovary, and round ligament were observed in the pelvis, while the left round ligament extended to the inguinal region, resulting in a clinical phenotype of pseudohermaphroditism. Case 7 was a 15-year-old raised as female. SNP array revealed that the MCs were derived from a pseudodicentric Y chromosome, with a karyotype of psu idic(Y)(p11.32) and approximately 38% 45,X mosaicism. Abdominal ultrasound showed a cord-like structure, suspicious for a uterine remnant.

Isodicentric Y chromosomes(idic(Y)) are unstable and often lost during mitosis, resulting in a predominant 45,X cell line and forming a mosaic 45,X/46,X, idic(Y) karyotype [31]. Although rarely detected prenatally, phenotypes of 45,X/46,X, idic(Y) patients range from Turner syndrome to disorders of sexual development, ambiguous genitalia, azoospermia, and intellectual disability, influenced by Y chromosome breakpoint locations, abnormal cell line proportions, and the presence or absence of *SRY* [32, 33]. In this study, a prenatal amniotic fluid sample (case 11) was found to carry an idic(Y) (Fig. 2, Case 11). SNP array analysis precisely mapped the breakpoint to Yq11.22. Approximately 25% of the cells exhibited an 18.1 Mb deletion spanning Yp11.31 to q11.22. This deleted segment encompasses the centromeric heterochromatin, the entire short arm(Yp), and the proximal portion of the long arm(Yq11.1 to q11.22), which contains 17 OMIM genes, including *SRY*,* USP9Y*, and *DDX3Y*. The *SRY* is the master regulator of male sex determination, and its deletion is associated with sex reversal. Mutations or deletions in *USP9Y* are linked to Y-linked spermatogenic failure. Notably, this deletion covers the azoospermia factor (AZFa) region. A complete AZFa deletion, which involves both *USP9Y* and *DDX3Y*, results in severe spermatogenic failure and azoospermia.

Furthermore, the SNP array identified an additional 7.9 Mb deletion in the Yq11.22q11.23 region. This second deletion, which falls within the AZFb+AZFc region, encompasses 21 OMIM genes, such as *CDY1*,* CDY2A*, and the *DAZ* gene family (*DAZ1*,* DAZ2*,* DAZ3*). Deletions in the AZFb and AZFc regions are a well-established cause of male infertility, typically manifesting as oligospermia or azoospermia.

Notably, the eventual clinical phenotype may not correlate with the proportion of abnormal cells detected in the amniotic fluid, as the karyotype of the fetal gonadal tissue could differ significantly from that of the cultured amniocytes. Hence, when a prenatal diagnosis reveals an idic(Y) chromosome, a comprehensive evaluation is essential. This should include not only molecular cytogenetic characterization but also detailed ultrasound assessment of fetal genital and gonadal development. Given the associated high risks of gonadoblastoma and infertility, and following extensive genetic counseling and informed consent, the parents opted for pregnancy termination.

In this study, MCs^T^ originating from X chromosome were confirmed by SNP array in cases 12 and 13. In case 12, SNP array accurately characterized the MC as a large deletion del(X)(q21.33q28), classified as pathogenic. In prenatal amniotic fluid, a ring MC mosaic with 45,X karyotype was detected in case 13, r(X)(p11.22q25). Turner syndrome patients with r(X) exhibit severe phenotypes including growth retardation, intellectual disability, and multiple malformations [34–36]. Case 13 was referred for prenatal diagnosis following a high-risk NIPT result suggestive of sex chromosome aneuploidy. Although prenatal ultrasound revealed no obvious fetal abnormalities, the potential for associated complications (such as disorders of sexual development and developmental delay) necessitates long-term postnatal follow-up. After genetic counseling and a detailed discussion of these risks, the parents chose to terminate the pregnancy.

### Genotype–phenotype correlation

Genotype–phenotype correlation in patients with MCs remains complex and highly variable. In many cases, the clinical outcome may be influenced by the genomic content of the duplicated chromosomal regions, including the presence of dosage-sensitive genes. Previous studies have suggested that copy number changes involving gene-rich regions may contribute to developmental abnormalities, whereas MCs containing heterochromatic or gene-poor regions are often associated with milder or absent phenotypes [37].

In addition, the clinical manifestations of MCs may vary depending on several factors, including the chromosomal origin of the marker chromosome, the size of the duplicated region, and the level of mosaicism. Similar variability has been reported in previously published cases, highlighting the difficulty of establishing clear genotype–phenotype correlations for these chromosomal abnormalities.

Therefore, although chromosomal microarray analysis provides valuable information regarding the genomic composition of MCs, further studies involving larger cohorts and detailed molecular characterization will be necessary to better understand the relationship between genomic alterations and clinical outcomes.

### Limitations

This study has certain limitations. First, only 15 cases of MCs were identified from a large cohort of 9,733 cytogenetic samples, resulting in a relatively small sample size for detailed genotype–phenotype correlation. Second, although SNP array was performed in seven cases, not all patients underwent molecular testing, which may have limited the comprehensiveness of the genomic characterization. Third, due to incomplete parental testing and follow-up data in some cases, it was difficult to fully clarify inheritance patterns and long-term phenotypic outcomes. Finally, as this was a single-center retrospective study, potential selection bias and limited generalizability should be considered. Fourth, it is acknowledged that SNP array has inherent limitations in detecting low-level mosaicism, and the mosaic status reported in this study is primarily based on cytogenetic findings [5]. Future multicenter studies with larger cohorts and more systematic molecular testing are needed to validate and expand these findings.

## Conclusion

In conclusion, MCs have diverse phenotypic outcomes. SNP array provides valuable insights into their origin and content, although complementary methods are required. An integrated diagnostic strategy is critical for accurate interpretation and effective genetic counseling, particularly in prenatal practice. However, the generalizability of our findings is limited by the small number of MC cases in this cohort. Future studies with larger sample sizes are needed to further elucidate the complex genotype-phenotype correlations.

## Data Availability

The datasets generated and/or analyzed during the current study are not publicly available due to patient privacy and ethical restrictions, but are available from the corresponding author on reasonable request.
